# Neonatal Resuscitation Research Priorities in Low- and Middle-Income Countries

**DOI:** 10.1155/2021/6938772

**Published:** 2021-11-25

**Authors:** Vivek V. Shukla, Somashekhar M. Nimbalkar

**Affiliations:** ^1^University of Alabama at Birmingham, Birmingham, Alabama, USA; ^2^Bhaikaka University, Karamsad, Gujarat, India

## Abstract

Several critical physiological changes occur during birth. Optimal and timely resuscitation is essential to avoid morbidity and mortality. The International Liaison Committee on Resuscitation (ILCOR) is a multinational committee that publishes evidence-based consensus and treatment recommendations for resuscitation in various scenarios including that for neonatal resuscitation. The majority of perinatal deaths occur in low- and middle-income countries (LMICs); however, there is limited research output from LMICs to generate evidence-based practice recommendations specific for LMICs. The current review identifies key areas of neonatal resuscitation-related research needed from LMICs to inform evidence-based resuscitation of neonates in LMICs.

## 1. Introduction

Extrauterine transition at birth and survival depends on several critical interlinked physiologic changes, including increase in systemic vascular resistance, decrease in pulmonary vascular resistance, closure of right to left shunts, alveolar fluid absorption and clearance, increase in metabolic demand, and endocrine changes [1–4]. The extrauterine transition in term newborns occurs spontaneously in about 85% [5], 10% need stimulation [6], 5% need positive pressure ventilation, 2% need intubation, and 0.1% need chest compressions [7, 8]. Cardiopulmonary resuscitation is needed for a relatively small proportion of newborns [7]; however, the absolute number is high when seen in the context of the total number of births [9]. Timely and appropriate resuscitation can prevent long-term morbidity and neonatal death [5, 10, 11].

## 2. Resuscitation Guidelines

The International Liaison Committee on Resuscitation (ILCOR) is a committee of 8 international resuscitation councils, the majority of which are from high-income countries, which publishes evidence-based consensus and treatment recommendations for neonatal resuscitation [12, 13]. The most recent guidelines are the 2020 International Consensus on Cardiopulmonary Resuscitation (CPR) and Emergency Cardiovascular Care (ECC) Science with Treatment Recommendations (CoSTR) for neonatal life support (NLS) with thorough evidence evaluation (Table 1) [14].

### 2.1. Perinatal Mortality in Low- and Middle-Income Countries

The global perinatal and neonatal deaths are estimated to be 4 million per year, and of that, about 98% occur in low- and middle-income countries (LMICs) [15–17]. Despite the sustained focus on reducing neonatal mortality by various international and local health organizations, neonatal deaths have increased in the relative contribution for under-5 child deaths [18]. Intrapartum asphyxia accounts for about 2 million stillbirths and neonatal deaths/year [17, 19–21] and results in long-term neurodevelopmental disabilities in about 1 million survivors/year [22] with a loss of about 41 million disability-adjusted life years/year [23]. Failure to initiate breathing after birth is the primary reason for death and disability in infants with birth asphyxia [24]. Lack of access to optimal resuscitation at birth is a preventable cause of death that is responsible for the majority of the perinatal and neonatal deaths in LMICs [10, 11, 25].

### 2.2. Resuscitation-Related Research in LMICs

The majority of resuscitation-related research that informs ILCOR neonatal resuscitation recommendations is from high-income countries. In the current ILCOR NLS-CoSTR, only 20 of a total of 96 of the referenced original science studies are from LMICs [14], and the majority of them are small sample size studies that have a limited grade of evidence. Interventional research can help identify the best management approach for a given disease or condition. This highlights the urgent need for enhancing resuscitation-related research for improving perinatal and neonatal outcomes in LMICs.

### 2.3. Why More Resuscitation-Related Research Is Needed from LMICs

The evidence from interventional research is specific for the environment and patient characteristics that it is tested and cannot be simply extrapolated to other settings. The research from high-income countries may not be applicable to low resource settings of LMICs because of differences in nutritional, environmental, socioeconomic, and genetic factors. Also, the ancillary resources and trained healthcare providers needed for a specific intervention may not be available. The application of the intervention without appropriate monitoring and follow-up may result in an increase in the risk of adverse outcomes. For example, several therapeutic hypothermia trials from high-income countries show benefits in survival and neurodevelopmental outcomes [26–28], establishing its role as a standard of care in infants with perinatal hypoxic-ischemic encephalopathy in high-income countries. However, as shown in the recently completed therapeutic hypothermia trial from LMICs, including infants from public sector neonatal units, infants in the therapeutic hypothermia group had increased mortality without any difference in severe disability in survivors [29]. This finding highlights the importance of validating research from high-income countries by evaluating them in LMICs before their routine adoption in LMICs. In order to successfully conduct interventional trials for neonatal resuscitation in LMICs, specific attention has to be directed at adequate funding for securing appropriate resources, training of healthcare personnel, and trial oversight for ensuring the protection of trial participants. After assessing the effectiveness of interventions, prior to adoption for a given setting, availability of adequately trained healthcare providers and availability of resources should be ensured, as resources and healthcare provider availability may differ between individual settings within LMICs [30–34]. Interventions should be carefully introduced while monitoring the responses for ongoing effectiveness evaluation and health policy insights needed for analyzing public health improvements and optimal resource allocation and utilization.

### 2.4. Areas of Resuscitation-Related Research Needing More Evidence from LMICs

Insufficient evidence from LMICs has led to knowledge gaps that are major barriers to the improvement of perinatal and neonatal outcomes in LMICs. There are several areas of neonatal resuscitation-related research that need better evidence from LMICs. Some of the research gaps include optimal oxygenation targets for resuscitation of preterm infants, therapeutic hypothermia for infants with perinatal depression from centers with higher levels of care, delivery of respiratory support at specific gestational age groups and testing of various interfaces, optimal thresholds, method, route, doses, and timing of various resuscitation-related interventions like chest compressions, epinephrine, volume expanders, and inotropes [14]. As outcomes of infants who are enrolled in interventional trials and the survivors getting admitted for intensive care may not be representative of the overall population, it is also essential to set up a collaborative registry of infants needing resuscitation at delivery from multiple institutes to better evaluate the outcomes of all infants with various conditions and those receiving different interventions. In addition to reporting hospital outcomes, efforts should be made to evaluate the long-term outcomes of infants enrolled in interventional trials, including 2-year neurodevelopmental and school-age outcomes using standardized assessment tools.

### 2.5. Future Directions

There needs to be a sustained effort towards improving the amount and quality of neonatal resuscitation-related research for informing evidence-based strategies from LMICs. Strengthening research infrastructure and supporting investigators with enhanced governmental and philanthropic research funding can help support quality neonatal research from LMICs. The creation of international, national, and interinstitutional research networks for collaboration, peer evaluation of research projects, and monitoring of ongoing projects can help streamline research efforts in LMICs. Efforts targeted towards training and mentoring early career investigators from LMICs can harness and promote the expertise and training needed for neonatal research.

## Figures and Tables

**Table 1:** 2020 CoSTR topics reviewed and evidence evaluation process [14].

Sr. no.	Topic	Subtopic	Process	Studies from LMICs
1	Anticipation and preparation	Prediction of need of respiratory support in the delivery room	Evidence update	No
		Effect of briefing/debriefing following neonatal resuscitation	Scoping review	No

2	Initial assessment and intervention	Warming adjuncts	Evidence update	No
		Suctioning of clear fluid	Scoping review	No
		Tracheal intubation and suction of nonvigorous meconium-stained newborns	Systematic review	Yes

3	Physiological monitoring and feedback devices	Heart rate monitoring during neonatal resuscitation	Evidence update	No

4	Ventilation and oxygenation	Sustained inflation	Systematic review	Yes
		Positive end-expiratory pressure (PEEP) versus no PEEP	Evidence update	No
		Continuous positive airway pressure (CPAP) versus intermittent PPV	Evidence update	No
		T-piece resuscitator versus self-inflating bag for ventilation	Scoping review	Yes
		Oxygen for preterm resuscitation	2019 CoSTR publication	No
		Oxygen for term resuscitation	2019 CoSTR publication	No

5	Circulatory support	CPR ratios for neonatal resuscitation	Evidence update	No
		2-thumb versus 2-finger compressions for neonatal resuscitation	Evidence update	No

6	Drug and fluid administration	Epinephrine (adrenaline) for neonatal resuscitation	Systematic review	No
		Intraosseous versus umbilical vein for emergency access	Systematic review	No
		Volume infusion during neonatal resuscitation	Evidence update	No
		Sodium bicarbonate during neonatal resuscitation	Evidence update	No

7	Prognostication during CPR	Impact of duration of intensive resuscitation	Systematic review	No

8	Postresuscitation care	Rewarming of hypothermic newborns	Evidence update	No
		Induced hypothermia in settings with limited resources	Evidence update	Yes
		Postresuscitation glucose management	Evidence update	No

## Data Availability

No original data was used in the current review.
